# Perceptions of osteoporosis risk assessment during menopause: a qualitative focus group study of patients and healthcare professionals in the UK

**DOI:** 10.1136/bmjph-2025-003892

**Published:** 2026-08-11

**Authors:** Claire Mann, Nai-Hao Yin, Helen Dawes, Marwan Bukhari, Jemma G Kerns, Frances Griffiths

**Affiliations:** 1 Lancaster Medical School, Lancaster University, Lancaster, UK; 2 Medical School, University of Warwick, Coventry, UK; 3 Medical School, University of Exeter, Exeter, UK; 4 NIHR Exeter BRC, Exeter, UK; 5 University Hospitals of Morecambe Bay NHS Foundation Trust, Kendal, UK; 6 Centre for Health Policy, University of the Witwatersrand Johannesburg, Johannesburg, South Africa

**Keywords:** Female, Community Health, Mass Screening, Risk Assessment

## Abstract

**Introduction:**

We conducted an exploratory, interpretive qualitative study to understand how UK patients and healthcare professionals perceive osteoporosis risk assessment around the menopausal transition and how they experience current care pathways.

**Methods:**

We conducted an exploratory qualitative study using focus groups to understand patient and clinician perceptions of osteoporosis risk assessment, associated needs and experiences and perceived barriers and opportunities within current care pathways. We interviewed 9 healthcare professionals (including general practitioners, pharmacists and other allied healthcare professionals) in focus groups of 2–3 and 10 patients in focus groups of 3–6. Each participant was involved in two focus group discussions about topics including the perceived need for osteoporosis risk assessment, diagnosis and prevention, exploring potential risk assessment pathways and considering the barriers and opportunities to developing risk assessment and diagnosis innovations for osteoporosis within the UK. Data reported in this paper were analysed using reflexive thematic analysis.

**Results:**

Three key themes emerged from this data: (1) public health literacy, (2) risk assessment and prevention and (3) opportunities for positive outcomes. The data demonstrate that low public health literacy, combined with high risk factors, presents significant opportunities for targeted interventions and improved outcomes in osteoporosis care, particularly around menopause. Future research should explore scalable models for delivering osteoporosis risk assessment in primary care settings, ensuring equitable access and sustainability within an overstretched system.

**Conclusions:**

This study reinforces longstanding evidence that osteoporosis remains under-recognised among perimenopausal women, despite elevated risk and decades of research. By contrast, the recent surge in menopause awareness presents a timely and strategic opportunity to embed osteoporosis messaging into existing public health discourse.

**WHAT IS ALREADY KNOWN ON THIS TOPIC**

- Patients are usually diagnosed for osteoporosis on an opportunistic basis, many after suffering a fracture or fall, adding to the vast costs of frailty on the UK National Health Service (NHS). Dual-energy X-ray absorptiometry (DEXA) scanning is the gold standard recommended by National Institute for Health and Care Excellence guidelines, but testing is expensive, and access is geographically varied. Early treatment and prevention of osteoporosis including lifestyle changes could save the NHS a significant amount of money and improve patient outcomes.

**WHAT THIS STUDY ADDS**

- As a result of this study, we understand the patient and clinician view on early risk assessment for osteoporosis. We understand patients experiences of suboptimal diagnosis and testing processes in the NHS and the system pressures and complications which impact patient care for bone health. We located a gap for early testing to prioritise patients for further testing and treatment.

**HOW THIS STUDY MIGHT AFFECT RESEARCH, PRACTICE OR POLICY**

- We have identified a need for further research to develop new methods for risk assessment and diagnosis and also into the health economics of osteoporosis risk assessment and prevention interventions, and into osteoporosis awareness and patient care postdiagnosis. The implications for practice are the potential to save money, prioritise patients for testing and improve patient outcomes. We identified some excellent support, especially via the Royal Osteoporosis Society, but also the need for further support and greater health promotion and awareness of osteoporosis in policy making.

## Introduction

 Osteoporosis is a public health burden in the UK^1^ and globally.^2^ The UK Royal Osteoporosis Society (ROS) demonstrated osteoporotic fractures cost the UK NHS and social care over £4.5 billion per year and this is predicted to rise to £6 billion by 2030 due to an ageing population. ROS also suggests that in the UK there are low levels of awareness of osteoporosis before fractures occur with 75% of patients unaware of risk levels and 52% who have never considered themselves as potentially at risk.^3^ The NHS 10-year plan commits to prevention and early intervention of osteoporosis through neighbourhood health centres, aligning with broader NHS goals to shift care from hospitals to communities.^4^

The British Menopause Society confirms that osteoporosis affects one in three menopausal women but is under-recognised.^5^ One UK report confirms that bone loss awareness and risk assessment often comes too late and many women are unaware of the increased risk of osteoporosis postmenopause before experiencing a fracture.^6^ One global study used a modified Osteoporosis Health Belief Scale to assess beliefs and perceptions and suggested that only 66% of menopausal women at risk were willing to have a DEXA scan due to lack of knowledge and misconceptions about osteoporosis.^7^

Conversely, there has been a recent increase of menopause awareness in the UK due to increasing media visibility and celebrity endorsements.^8–10^ Between 2015 and 2023, UK Hormone Replacement Therapy (HRT) prescriptions rose by 47%,^11,12^ a trend likely to continue following the introduction of an HRT prepayment certificate in April 2023, reducing the overall cost of the medicine.^13^

For several decades there has been research evidence that risk of osteoporosis increases in later life and affects females significantly more than males.^14,15^ Studies have shown that this is likely to be due to the reduced ovarian oestrogen after menopause causing a significant loss in bone strength.^16^ The large-scale longitudinal US SWAN study confirms that bone mineral density (BMD) declines substantially in the late perimenopause through to postmenopause, reinforcing the critical role of hormonal changes in osteoporosis risk.^17^

Recent increases in menopause-related healthcare engagement, including rising HRT consultations, indicate that more women are seeking support during the menopausal transition. This increased contact provides a practical opportunity to integrate proactive osteoporosis risk assessment into routine care. As women navigate decisions about symptom management and midlife health, menopause consultations may serve as an acceptable touchpoint for introducing bone-health information, raising awareness of fracture risk and embedding tools such as FRAX or QFracture.

Current UK National Institute for Health and Care Excellence (NICE) guidelines recommend assessing fracture risk according to previous fractures, use of steroids, history of falls or other factors such as family history or low body mass index.^18,19^ Guidelines recommend use of a risk assessment tool (FRAX or QFracture) followed by a DEXA scan for diagnosis. Globally DEXA (X-ray bone density scanning) is considered the gold standard in osteoporosis risk measurement and management.^20–22^ DEXA was introduced in the 1980s and from 1994 WHO defined osteoporosis based on a DEXA measurement. DEXA has been routinely used in hospitals for diagnosis. FRAX is an internet-based big data model that calculates the 10-year probability of a major osteoporotic fracture derived from the incidence of fractures as recorded in large cohorts including randomised controlled trials (RCTs). The FRAX tool is globally effective as it has been adapted and calibrated to the epidemiology of fracture and death in different countries so gives the option of country-specific calculations.^23,24^ QFracture is a UK-developed, web-based risk prediction algorithm designed to estimate the 10-year probability of osteoporotic and hip fractures. It is derived from anonymised primary care data from over 2 million patients in the QResearch database, making it highly representative of the UK population. Unlike FRAX, QFracture does not require BMD input and incorporates a broader range of clinical risk factors including falls, smoking, alcohol use, comorbidities and medication history and is integrated into many UK general practitioner (GP) systems.^25^ The limitation is that both FRAX and QFracture have a minimum age recommendation (40+/35+).

There are a number of studies that focus on osteoporosis diagnosis and treatment for women around menopause.^26–28^ One large-scale investigation into postmenopausal women aged 50–64 years showed that osteoporosis is prevalent in this group (15.4%) and that risk assessment tools are effective in identifying those at risk.^29^ A 2024 study demonstrated that low BMD is a strong predictor of fracture risk, particularly in postmenopausal women.^30^ The study highlights the importance of early risk assessment in women due to their higher susceptibility to osteoporosis compared with men. One study suggests that the probability of future fractures could be calculated by identifying risk factors in all menopausal women.^31^

HRT is the only known effective pharmacological treatment in use for osteoporosis prevention, recommended by the Society for Endocrinology.^32^ However, the Women’s Health Initiative in the early 2000s identified a link between HRT use and breast cancer which had a significant impact on reducing or limiting HRT prescribing.^33^ Further treatments have been developed including bisphosphonates and confidence in HRT has been restored over time.^34^ NICE currently recommends HRT for bone protection in women under 60 in the UK.^35^ Conceptually, menopause consultation represents an opportunity for brief risk communication/assessment (eg, FRAX/QFracture^23,24^ prompt) which offers the chance for prevention actions and/or referral.

Evidence demonstrates that prevention or delay of onset of osteoporosis is possible through changing lifestyles in particular diet and exercise.^18,30^ A recent review emphasises that non-pharmacological interventions such as a balanced diet rich in calcium and vitamin D, regular weight-bearing exercise, and avoiding smoking and excessive alcohol can play a significant role in preventing further bone loss. These lifestyle changes support bone health and can complement pharmacological treatments.^36^

While both in the UK and globally, awareness of risk has not improved over the last 25 years, by contrast there is evidence of a wave in improving menopause awareness, diagnosis and treatment. We are interested to understand if this rise in awareness of menopause represents an opportunity to increase awareness of osteoporosis and who is at risk. This paper reports on the findings of a qualitative study exploring the acceptability of osteoporosis risk assessment, screening and diagnosis to patients and healthcare professionals (HCPs) in the UK. Our aim was not to test predefined hypotheses; rather, we sought to generate in-depth insights into osteoporosis awareness, barriers to assessment, and workflow touchpoints to inform service development and patient outcomes.

## Methods

### Aim and design

This qualitative study adopted an interpretive, pragmatic qualitative approach to understand how patients and HCPs perceive osteoporosis risk assessment, diagnosis and prevention, and how they experience current care pathways. Our aim was not to test predefined hypotheses, but to generate in-depth insights into perceived needs, barriers, and opportunities relating to bone health and osteoporosis diagnosis during the menopausal transition. Focus groups were selected to support the co-construction of knowledge across shared experiences. Reporting follows the Consolidated criteria for Reporting Qualitative research (COREQ) checklist (checklist provided as online supplemental file).

Although rooted in an interpretivist concern for how participants construct meaning, our analysis also drew on a pragmatic orientation that allowed incorporation of both inductive insights and deductive codes linked to preidentified areas of interest. This mixed approach acknowledges that qualitative health research often balances exploration of lived experience with targeted inquiry driven by applied aims.

We conducted focus groups both with patients with experience of osteoporosis and separately with clinicians who treat patients at risk of osteoporosis. We aimed to recruit 10 patients and HCPs to represent a broad range of experiences but stopped recruiting according to our short timescales for the project at 9 HCPs and 10 patients.

HCPs were recruited via local clinical networks and snowballing. Patient and public involvement (PPI) participants were recruited via the ROS research route.

### Participants and setting

Patient eligibility included those registered with ROS with an interest and understanding of bone health who either (a) had a diagnosis of osteoporosis or (b) self-identified as at risk of osteoporosis. We sought a broad sample of age, UK location and time from diagnosis. The patient sample intentionally included both individuals with an established osteoporosis diagnosis and individuals who self-identified as ‘at risk’. This approach reflected our aim to explore experiences across the continuum of risk perception, assessment and diagnosis. The patient sample included one male participant with a diagnosis of osteoporosis. This reflects our broader sampling aim to capture perspectives across the continuum of osteoporosis experience, beyond menopause-specific pathways.

For diagnosed participants, time since diagnosis ranged from recent (<1 year) to several years, capturing variation in how people engage with ongoing bone-health information and services. HCP eligibility included any qualified HCP with ≥1 year of relevant experience in frontline care of bone health. Osteoporosis risk assessment and advice are delivered across multiple points in primary care, including general practice, physiotherapy, community pharmacy and diagnostic imaging. To reflect this distributed care model, we intentionally recruited clinicians from diverse professional roles (GPs, practice nurses, physiotherapists, pharmacists and radiographers). This enabled us to explore how responsibilities for identifying, assessing and advising on bone health are understood across the wider healthcare ecosystem. Exclusions were inability to participate in group discussion, cognitive impairment affecting consent or insufficient English for qualitative engagement.

Data were collected from 10 patients and 9 HCPs in focus groups of 2–3 (HCPs) and 3–6 (patients). Focus-group sizes for HCPs were smaller than typical (2–3 participants) due to substantial scheduling limitations and clinical workload constraints, which restricted simultaneous availability. Small groups are recognised in qualitative research as still capable of producing rich data, particularly when participants share professional grounding. Patient focus groups included 4–6 participants, consistent with guidance for generating diverse perspectives while allowing depth of discussion. Each participant was involved in two focus group discussions about topics including the perceived need for osteoporosis risk assessment, diagnosis and prevention, exploring potential risk assessment pathways and considering the barriers and opportunities to developing risk assessment and diagnosis innovations for osteoporosis within the UK. Participants were invited to attend both rounds (one workshop per round), although some attended only one due to availability. In total, two PPI participants and three HCP participants attended only one round due to limited availability or non-response to follow-up invitations. Our participants were predominantly females, reflecting the higher proportion of females experiencing osteoporosis (see tables 1 and 2).

**Table 1:** HCP participants

Label	Clinical role/expertise	Region	Gender	Rounds
HCP-1	GP	East Midlands	Female	1, 2
HCP-2	Pharmacist	East Midlands	Female	1, 2
HCP-3	Physician associate	West Midlands	Female	1
HCP-4	GP	West Midlands	Male	1, 2
HCP-5	Pharmacist	London	Female	1, 2
HCP-6	Muscoskeletal (MSK) physiotherapist	South East	Female	1, 2
HCP-7	GP	West Midlands	Female	1, 2
HCP-8	Radiographer	South West	Female	1, 2
HCP-9	Radiographer	South West	Female	2

GP, general practitioner; HCPs, healthcare professionals.

**Table 2:** PPI participants

Label	Diagnosis experience	Region	Gender	Age range	Rounds
PPI01	6 years postdiagnosis	East of England	Female	70–79	1, 2
PPI02	30 years postdiagnosis	London	Female	60–69	1
PPI03	5 years postdiagnosis	West Midlands	Female	50–59	1
PPI04	4 years postdiagnosis	South West	Female	70–79	1, 2
PPI05	4 years postdiagnosis	South East	Male	70–79	1, 2
PPI06	20 years postdiagnosis	South West	Female	60–69	1, 2
PPI07	2 years postdiagnosis	East Midlands	Female	50–59	1
PPI08	14 years postdiagnosis	Scotland	Female	70–79	1, 2
PPI09	6 years postdiagnosis	North East	Female	60–69	1, 2
PPI10	At risk (breast cancer, medical menopause) Under 3-year monitoring/watchful waiting	East Midlands	Female	30–40	1, 2

PPI, patient and public involvement.

### Development of the focus group guides

The focus-group topic guides were developed collaboratively by the multidisciplinary research team and a PPI representative. Its content was informed though a combination of existing literature on osteoporosis risk assessment, menopause-related health behaviours, and barriers to screening uptake and clinical expertise within the team in general practice, physiotherapy and women’s health. Initial draft questions were structured to elicit perceptions, experiences and decision-making related to bone health and care pathways. The guide underwent iterative refinement through team discussion to ensure conceptual clarity and neutrality of prompts. It was piloted with one clinician and one member of the public group to assess comprehension, flow and timing, leading to minor adjustments in ordering and phrasing. The final guide maintained flexibility to allow emergent issues to be explored while ensuring coverage of core domains relevant to the study objectives. The complete topic guide is provided as online supplemental file 1 to support transparency and reproducibility.

Workshop and Focus-Group Structure: The data-collection process comprised two rounds, each containing a PPI workshop and an HCP workshop. Participants were invited to attend both rounds; group composition varied between rounds due to scheduling, and participants were allocated to a single workshop per round (one PPI and one HCP group per round).

Focus groups were conducted separately for patient/public participants and HCPs. This separation was intentional to facilitate open and candid discussion. Keeping groups homogeneous in terms of participant role helped minimise power dynamics or hierarchy effects (eg, patients feeling inhibited in the presence of clinicians, or junior clinicians feeling constrained around senior staff). It also allowed participants to speak freely about experiences of accessing or providing osteoporosis risk assessment without concern for judgement or professional sensitivity. We did not conduct mixed patient–HCP groups because the purpose of this study was to explore experiences, perceptions and needs rather than co-design solutions. Separate groups enabled us to examine how understandings of osteoporosis risk assessment differ across roles before considering points of convergence. Round 1 explored experiences, perceptions of fracture risk and views on current pathways. Round 2 revisited these topics to deepen understanding and facilitators did not introduce interim findings to avoid shaping contributions. Most participants attended both rounds; a minority attended one due to availability. Each workshop lasted 55–90 min, generating around 9 hours of discussion overall. All workshops took place online and CM facilitated all workshops, N-HY and JK provided technical/observational support on selected sessions. Structured prompts, balanced turn-taking and active monitoring were used to mitigate dominance and support equitable participation. Table 3 outlines the data collection process elements.

**Table 3:** Data collection process elements

Element	Description
Total number of sessions	4 workshops: 2 PPI (round 1 and round 2) and 2 HCP (round 1 and round 2).
Separate versus mixed	PPI and HCP workshops conducted separately (no mixed groups).
Purpose of each round	Round 1: explore experiences, perceptions and current pathways. Round 2: revisit and deepen understanding.
Group sizes	PPI: 4–6; HCP: 2–3 (reflecting clinical availability)
Group formation	Based on availability; composition varied between rounds; no participant attended two groups within the same round. PPI and HCP groups were conducted separately to minimise hierarchy and encourage candid discussion.
Attendance patterns	Most participants attended both rounds; a small minority attended once due to availability.
Session duration/total hours	55–90 min per workshop; around 9 hours total discussion time.
Facilitator and dynamics	CM facilitated all workshops; N-HY and JK provided technical/observational support and co-facilitation on selected sessions. Techniques used included structured prompts/turn-taking used to minimise hierarchy/dominance.

HCP, healthcare professional; PPI, patient and public involvement.

All focus group data were collected online to facilitate the inclusion of people geographically across the UK by one researcher (CM) supported by others on individual occasions (N-HY, JK). First workshops (round 1) were held in November 2024–January 2025 and second workshops were held between May and July 2025 (round 2). Interviews were audio recorded, transcribed verbatim, anonymised and transferred to NVivo V.14 for coding.

### Data analysis

We conducted reflexive thematic analysis (RTA) following Braun and Clarke’s six-phase approach.^37–39^ Analysis began with familiarisation by all team members, followed by inductive, semantic coding by one researcher (CM). Transcripts were managed in NVivo V.14 to support coding and analytic memoing. Codes were treated as flexible analytic prompts rather than a fixed hierarchy and themes were developed interpretively across the dataset. Consistent with RTA, we did not pursue coding consensus or reliability metrics. In line with RTA, we did not seek coding reliability or consensus, and analytic interpretation was developed through reflexive engagement with the data. Reflexivity served as an analytic resource: researchers kept reflexive memos and held iterative team discussions to surface assumptions and extend interpretation. Although coding and theme development were inductive, the discussion draws on the ‘Capability-Opportunity-Motivation enables Behaviour’ (COM-B) framework to contextualise how barriers and opportunities identified in analysis relate to determinants of engagement in preventive care. Throughout, we use the term RTA to distinguish this approach from other forms of thematic analysis.

### Reflexivity and positionality

No prior relationship existed between the researchers and participants before recruitment. Participants were informed about the purpose of the study, the researchers’ roles, and the general aims of the project via the participant information sheet and verbal introduction.

Workshops were facilitated by CM (female), who holds academic expertise in women’s health and a commercial role as CEO of a menopause-focused digital health company. We recognise that these intersecting identities may have shaped question framing and sensitivity to opportunity narratives. Topic guides were co-developed within the wider team, including members without commercial interests; a subset of transcripts was reviewed independently to check for overemphasis on commercially aligned themes; and reflexive dialogue was used to challenge assumptions.

### Patient and public involvement

A PPI contributor supported the development of this study. The PPI contributor reviewed and piloted the focus group topic guide, providing feedback on question clarity. This input informed refinements to the wording of the question prompts. Patient participants were actively engaged as contributors within the focus groups shaping the data generated through discussion of their lived experiences. Findings may inform future patient-facing materials and service development.

## Results

Three key themes emerged from the data: (1) public health literacy, (2) risk assessment and prevention and (3) opportunities for positive outcomes. To enhance analytic clarity, each overarching theme is presented with subthemes. These subthemes helped articulate distinctions and strengthen thematic boundaries while remaining consistent with an inductive, interpretive approach. We present three overarching themes, each now supported by clearly defined subthemes to illustrate variations in experience and delineate thematic boundaries. Where relevant, we differentiate between PPI and HCP perspectives and highlight areas of convergence and divergence. Where helpful for interpretation, we differentiate between at-risk and diagnosed participants and note where time since diagnosis shaped perspectives (eg, confidence navigating care, expectations of assessment or emotional responses to fracture risk). Where relevant, we outline differences between professional groups to illustrate how roles, responsibilities and workflow shape perspectives on osteoporosis risk assessment.

### Public health literacy about menopause and osteoporosis

#### Fragmented information pathways

Participants described inconsistent sources and uncertainty about risk factors and thresholds for concern. Women with recent diagnoses tended to describe heightened uncertainty and urgency, while those at risk described a more speculative, anticipatory relationship to information and assessment. HCPs and patients described a public lack of awareness understanding about the relationship between menopause and osteoporosis.

In all patient groups, some participants were unaware that menopause marked a period of increased risk,

I didn’t realise that the menopause was a period of greater danger, and I should have been more aware. (PPI02)

Some women had not been informed about benefits of taking HRT on bone health which had impacted their treatment choices.

I had HRT, which for quite a negligible reason, the GP stopped giving me in 2018… I’m going back on HRT… (PPI07)

Awareness of osteoporosis as a women’s health issue was low in the patient groups. Most participants had only become aware of their risk after a fracture or because of a family history. Patients were rarely aware of osteoporosis before their experience of diagnosis and many suggested they had friends who would not be aware of osteoporosis risks or treatments at all.

By contrast most HCPs in the groups identified menopause as a risk factor for osteoporosis

Female patients, if they enter …menopause … they’re gonna be high risk. (HCP01)

Patients who are entering perimenopause or menopause [are at risk] (HCP05)

Some HCPs suggested that not all those working in primary care are fully informed about osteoporosis. One clinician suggested a general low level of understanding of the risk factors for osteoporosis within community pharmacies.

Participants in all groups suggested clearer public health information and better integration of menopause-related risk into osteoporosis education and care pathways would be helpful. Patients suggested that low awareness of menopause had changed recently and there was potential to similarly elevate awareness of osteoporosis

It’s like the menopause used to be, it was ‘just get on with it’. (PPI03)

#### Osteoporosis as an older person’s disease

Many patient participants conceptualised osteoporosis as a condition that primarily affects ‘older women’ or ‘frail, elderly people’. This framing risk perception: perimenopausal and younger postmenopausal women often did not view themselves as at risk, which reduced urgency to seek assessment or engage with preventive behaviours. Some at-risk participants described being ‘shocked’ to receive advice about bone health because they associated osteoporosis with older people. Patients suggested that the image of osteoporosis as associated with elderly patients was a potential barrier to raising awareness.

I had no idea at all what any signs of osteoporosis was. There’s none in my family. All I’ve seen is old ladies, my age, bent double. But no indication of how you acquired it or what you could do to prevent it at all. (PPI01)

Several patients suggested that having a risk assessment programme for osteoporosis would have the potential to raise widespread awareness of bone health, especially if targeted at an audience of women in perimenopause.

I think some sort of test like this for osteoporosis would raise awareness because is it over 50% of women will have it… (PPI03)

HCPs reported difficulty shifting this perception in time-limited consultations. Clinicians noted that menopause and HRT discussion might be missed opportunities for raising further awareness.

I do a lot of menopausal talks… menopause is probably one of the contributing factors… (HCP03)

There was consensus that public health awareness was low and public health and primary care messaging should better connect menopause and osteoporosis risk for the public.

### Risk assessment and prevention in menopause

#### Unclear responsibility for initiating assessment

Patients often assumed health services and clinicians would proactively offer assessment. By contrast HCPs varied on who should initiate conversations about bone health. Menopause was identified by both groups of participants as a critical window for risk assessment throughout the HCP discussions.

So, I would say … menopause patients who perhaps would benefit from these tests and the screening tools… (HCP01)

Clinicians saw menopause consultations as an opportunity to address bone health; several patients, however, reported that these consultations focused almost exclusively on vasomotor symptoms or HRT, with little or no discussion of bone health.

#### Structural capacity constraints in primary care

Workload pressure and limited time curtailed opportunistic prevention, even where motivation existed. HCPs consistently described primary care as operating under substantial workload pressure, which limited their ability to offer proactive osteoporosis risk assessment or opportunistic preventive conversations. GPs highlighted the tension between competing consultation priorities and the absence of protected time for preventive care. Pharmacists and physiotherapists described wanting to support bone-health education but noted that workflow pressures and contractual obligations limited opportunities. These constraints functioned as a cross-cutting structural barrier, shaping not only clinicians’ capacity to act but also patients’ perceptions of access and eligibility.

Although most HCPs identified menopause as a risk patients suggested they had not been given access to this information.

Some patients understood the risk of osteoporosis for women approaching menopause. Patients felt that current services disproportionately targeted older, postfracture populations, missing opportunities for prevention:

They would probably only pick people who had gone through the menopause… or had surgery, cancer, and not able to take HRT. (PPI06)

Across both patient and clinician accounts, capacity constraints in primary care surfaced repeatedly as a structural barrier that constrained opportunities for timely risk assessment, limited access to credible information, and shaped expectations about who would initiate preventive conversations.

#### Navigating access and eligibility

All primary HCPs were aware of the FRAX tool but most had limited time to use it in primary care practices. Confusion persisted around screening, how/when FRAX or QFracture are used and what triggers DXA referral. Participants diagnosed several years earlier often recounted fragmented follow-up experiences, contrasting with at-risk participants who expressed uncertainty about where to begin.

One female patient reported that she struggled to access diagnosis and treatment because she was not menopausal and therefore considered low risk.

They said it was impossible to be osteoporosis… it took about a year to get a DEXA scan. (PPI02)

Some patients suggested there was a need to target risk assessment earlier, at women in their 40s and 50s.

I think screening would be a good idea for set groups… like people with the perimenopause… (PPI01)

Patients recognised that they had high levels of awareness of osteoporosis based on their own personal experiences and that further work should examine perceptions of osteoporosis risk in perimenopausal women without any experience of risk assessment.

I’ve got a lot of friends that are just at that stage where they’re … thinking they might be going through the perimenopause at the moment… you might just assume that [osteoporosis risk] is not going to be an issue for you at that point. (PPI10)

Perimenopausal women were the most popular cohort which participants suggested that impact could be made through risk assessment and preventative action.

### Opportunities for positive outcomes

Patients framed opportunities in terms of clearer pathways and trusted guidance, while clinicians focused on workflow-feasible prompts and system integration.

#### Menopause as a meaningful touchpoint

Menopause/HRT consultations were viewed as acceptable entry points for brief risk conversations. Clinicians noted that menopause-related bone loss is often hinted at by patients in menopause conversations.

If you know a 45-year-old who could be a female or who could be going having perimenopausal symptoms normally does ask ….’Do you think my bones are weak now?’ or ‘I just have these horrible aches and pains here and there’ you know, they have those questions. (HCP03)

The emotional and physical toll of osteoporosis was described as significant, particularly when diagnosis came late. Several participants expressed regret that earlier risk assessment, especially around menopause might have changed their behaviours and therefore trajectory.

What type of population would [be] most helped? That is the people who are obviously pre diagnosis who haven’t fractured anything, but …are at risk, although they didn’t realise it. Those are the types of people (PPI09)

#### Workflow-aligned prevention opportunities

Clinicians agreed that osteoporosis could be under-addressed in primary care and that risk assessment embedded within menopause appointments could target awareness raising. However primary care physicians noted that they are currently struggling to provide mainstream services and as such risk assessment and opportunistic health prevention opportunities are limited.

I think if you’re not working in GP at the moment you don’t understand the pressures that GPs are under … we are on our knees. (HCP01)

GPs often framed risk assessment as competing with other consultation priorities, whereas pharmacists described frequent informal conversations with midlife women that could serve as potential touchpoints for educational messaging.

HRT was mentioned by both patients and clinicians as a potential preventative measure to help bone strength. The discussions with patients about HRT were proposed as an opportunity to mention bone health and consider bone health risk assessment with a potentially receptive population.

I think this is probably going to help people who are more … interested in their own health so possibly …the ladies who are on HRT … and actually they probably should because they’re going to be more prone to osteoporosis. (HCP08)

Clinicians suggested that patients seek more preventative health advice as they age.

I think in general the population in general when they get to a certain age, they do start to ask for things to do, bone health or you know any kind of preventative things (HCP02)

They also suggested that some patients at risk of osteoporosis have the wrong information about lifestyle changes and the impact on bone health risk

I think there’s a lot of a lot of myths and misinformation about there. So a lot of patients are quite fearful about doing vigorous exercise and are worried that it might cause damage. And so it takes a lot of education and reassurance that they should be exercising and exercising hard to have a positive impact on their own health (HCP06)

Menopause appointments and HRT prescribing were identified as potential opportunities to discuss bone health and screening.

#### Trust, communication and motivation

Short, credible messages improved willingness to act among patient participants. Several patient participants described making lifestyle changes following diagnosis, including changes to diet, exercise and medication.

I’ve completely changed my fitness programme and my diet… I’m going back on HRT. (PPI02)

Patients and HCPs suggested that although risk assessment and awareness were important, patients would have to be motivated to make positive behaviour changes that might impact their health. Some female patients suggested that since risk assessment and in particular since diagnosis they had been motivated to make positive lifestyle changes.

I do a lot of Zumba, Pilates… My DEXA scan was stable, what I’m doing seems to be working. (PPI09)

Several patients suggested that changes to their body as a result of menopause and declining bone health had inspired them to make positive lifestyle changes, but there were limited resources or expertise in the community to support them.

Most gyms… staff don’t have a clue that one in two women over 50 will break a bone due to osteoporosis. (PPI09)

Several participants reported unsuccessful attempts to access physiotherapy services with specialist expertise in menopausal bone health, and expressed the need for better support services to inform healthy behaviour change.

Both patient and clinical participants suggested that the ideal audience for osteoporosis risk assessment would be those young enough and motivated enough to take preventative action.

I think it would certainly change the experience of people in the future because they could be put on the drugs at an earlier stage rather than people like us that have already suffered the fractures or problems before we were put on the medication. (PPI04)

Perimenopause was identified by all participants as a mid-life trigger that might drive a patient into primary care representing an opportunity to raise awareness of risk and provide guidance about prevention.

The data demonstrate that low public health literacy, combined with high risk factors, presents significant opportunities for targeted interventions and improved outcomes in osteoporosis care, particularly around menopause.

## Discussion

Menopause presents an opportunity for osteoporosis awareness and assessment demonstrated through three patterns in the data presented. Osteoporosis is often perceived as an older person’s disease reducing perceptions of risk, menopause brings increased healthcare contact (eg, symptom/HRT consultations) when women are motivated to engage and clinicians identified workflow-compatible points where brief risk prompts and signposting could realistically occur despite capacity constraints.

Our study supports evidence from the literature that awareness of osteoporosis remains low prior to diagnosis or fracture.^1–3^ Despite over 25 years of research highlighting the elevated risk of osteoporosis among menopausal women,^26–30,40,41^ our findings indicate that public understanding and clinical engagement with this risk have remained largely unchanged. This persistent gap suggests missed opportunities for early risk assessment, intervention and preventative care. While patients emphasised confusion and unmet informational needs, clinicians identified system-level capacity constraints and uncertainties about role boundaries. These differing vantage points highlight the importance of designing interventions that reconcile both experiential and structural barriers

Our study reinforces evidence of a recent surge in menopause awareness in the UK,^8–11^ as both patients and clinicians highlighted its relevance to osteoporosis risk assessment and diagnosis. This presents a strategic opportunity to align menopause and osteoporosis messaging, an approach which has previously been explored but not fully capitalised on.^28–30^ Analogous public health campaigns have successfully piggybacked related conditions to amplify impact. For example, initiatives linking obesity with type 2 diabetes have improved risk identification and lifestyle modification.^42,43^ These precedents offer a compelling framework for integrating osteoporosis messaging within menopause awareness efforts.

Clinical awareness of osteoporosis risk has been demonstrated in the literature over a long period of time.^1–3,6,15–17^ Historical studies also highlight attempts at preventative intervention in this cohort, underscoring a long-standing recognition of the issue.^42–45^ Despite being aware of the risk of osteoporosis for women at menopause, clinicians (GPs in particular) reported that they currently did not have capacity to undertake opportunistic prevention consultations for menopausal women^46^. These findings suggest that systemic barriers, rather than clinical awareness, may be the current primary obstacle to early intervention. Increased healthcare contact during the menopausal transition, including consultations focused on symptom management and HRT decision-making, may represent a practical touchpoint for preventive action. Conceptually, these encounters provide an opportunity for clinicians to introduce information about bone health, initiate or offer osteoporosis risk assessment using tools such as FRAX or QFracture and support preventive behaviours or referral where appropriate. Our qualitative study explores how patients and HCPs perceive this potential pathway, and whether menopause-related consultations are viewed as acceptable and feasible moments for integrating osteoporosis prevention into routine care.

The subthemes highlight the patterned distinctions within each domain, allowing clearer implications for designing targeted, pragmatic interventions across primary care roles. Feasible strategies suggested from our study include pre-consultation prompts (online triage or previsit checklists to flag risk factors), brief standardised scripts to deliver consistent bone-health messages, delegation of opportunistic prevention to pharmacists/nurses/muscoskeletal (MSK) clinicians, screening programmes for patients at risk and clearer referral pathways for DXA and FRAX/QFracture follow-up. Within time-limited practice, HRT consultations can cue a brief FRAX/QFracture check, clarify key risk factors, and signpost to DXA where appropriate. Not all menopausal women seek HRT or menopause-focused care; HRT should be viewed as one of several multiprofessional touchpoints (eg, pharmacy, medication reviews, MSK/rehab).

Our patient data revealed that many individuals took proactive steps to address their osteoporosis risk following risk assessment through both medication and lifestyle changes. These behaviours are consistent with findings from qualitative studies conducted over the past 25 years^32–34^ and align with the notable rise in HRT prescribing linked to increasing menopause awareness in the UK.^7–9^ Such evidence suggests that women respond positively to health messaging and are motivated to engage in preventative action. Targeting perimenopausal communities with timely education and risk assessment interventions may therefore foster earlier behaviour change and reduce long-term osteoporosis risk.

Women’s Health Hubs, as outlined in the UK Government’s Women’s Health Strategy, represent a new model of integrated care designed to improve access, experience, and outcomes for women across the life course. These hubs bring together services such as contraception, menstrual health, menopause care and cervical screening and are increasingly being delivered in community settings, including GP practices and virtual platforms. Emphasis on preventive care and intermediate-level services positions these hubs as a promising setting for osteoporosis risk assessment and early intervention, particularly for perimenopausal and postmenopausal women.^41^

Our findings support the longstanding hypothesis that low public awareness, combined with high risk, makes the perimenopausal stage a critical window for osteoporosis prevention. Although this insight has been documented over the past 25 years, aside from recent gains in menopause awareness and HRT uptake, meaningful change has been limited. Introducing a targeted risk assessment programme could drive earlier intervention and health-promoting behaviour, with potential long-term cost savings for the NHS. Hearing that one is at risk can act as a powerful cue to action, as described in the Health Belief Model, which posits that individuals are more likely to engage in health-promoting behaviours when they perceive a serious threat and believe that taking action will reduce that threat. This aligns with the 2025 NHS 10-Year Plan, which prioritises a shift toward preventative care.^41^ However, we recognise that current capacity constraints in primary care may pose challenges to implementing such interventions at scale.

Although our analysis was inductive, the findings align with COM-B elements.^39^ Reframing the perception of osteoporosis as an older person’s disease targets psychological capability, increasing appointment-time prompts expands physical opportunity, and brief scripts/credible messaging support motivation for preventive action. Our findings illustrate how menopause-related consultations, including those centred on HRT decision-making, can provide meaningful micro-opportunities for clinicians to initiate bone-health conversations. These encounters appeared to align psychological capability (awareness), physical opportunity (clinical contact) and motivation (concerns about midlife health), reinforcing menopause as a practical site for integrating osteoporosis risk assessment within routine care pathways.

Future research should explore theoretically based, scalable models for delivering osteoporosis risk assessment in primary care settings, ensuring equitable access and sustainability within an overstretched system. While DEXA remains the gold standard for diagnosing osteoporosis, its use as a population-level screening tool has been dismissed by the UK National Screening Committee due to limited impact on fracture outcomes and cost-effectiveness. This highlights the urgent need for the development and validation of alternative tools that are both clinically effective and feasible for use in community settings. Risk prediction algorithms such as FRAX and QFracture offer promising approaches, but further innovation is needed to enhance early identification and preventive intervention, particularly targeting women approaching menopause.

Given our small, self-selected sample and pragmatic recruitment, we do not claim representativeness; instead, we offer transferable prompts for service design that require testing in larger, purposively sampled studies.

### Limitations

This study has several methodological limitations. Our small, self-selected sample were recruited via clinical networks and a disease-interest route which both introduces potential selection bias and potentially limits demographic diversity. We included one male participant in the PPI sample, which does not align directly with the menopause-focused framing of the study. While this provided some broader contextual insight into osteoporosis experiences, his data were not central to the development of menopause-related themes.

Although safeguards were implemented, we acknowledge that CM’s dual academic–commercial position may have shaped aspects of questioning and interpretation. Reflexive awareness and team-based analytic dialogue were used to mitigate this influence, but readers should consider these positionalities when interpreting findings related to ‘opportunity’ narratives.

Clinician group sizes were small due to workload constraints, which may have limited interactional dynamics. Although steps were taken to minimise cross-round contamination including changing group compositions and withholding interim analytic insights, we acknowledge that individual reflection between rounds may have shaped participants’ later contributions. This is an inherent feature of longitudinal qualitative work and was considered analytically. Although multiprofessional sampling enabled us to capture diverse perspectives across primary care, differences in clinical remit and workflow may influence how each group interprets opportunities for assessment and prevention. While we attended to these distinctions analytically, some profession-specific nuances may warrant deeper exploration in future studies.

We also recognise that including both diagnosed and at-risk participants within patient groups may have shaped patterns of disclosure, although facilitation strategies were used to support equitable contribution.

Although we used iterative analysis and noted when no new codes were emerging, we did not apply a formal saturation procedure and findings should be read as prominent patterns rather than an exhaustive account. Our analytic stance was pragmatic-interpretivist, combining inductive insights with a priori interests in service opportunities, which may introduce tensions between open-ended exploration and theory-informed interpretation.

These factors should be considered when interpreting the transferability of the insights.

## Conclusions

This study reinforces longstanding evidence that osteoporosis remains under-recognised among perimenopausal women, despite elevated risk and decades of research. In contrast, the recent surge in menopause awareness presents a timely and strategic opportunity to embed osteoporosis messaging into existing public health discourse. Our findings suggest that while clinical knowledge of osteoporosis risk is well established, systemic barriers within primary care continue to limit preventative action. Patient responses to understanding risk underscore the potential for positive behavioural change when awareness is raised, with clear parallels to other successful public health campaigns such as diabetes and obesity prevention. Targeted risk assessment and education during the perimenopausal stage could therefore act as a catalyst for healthier ageing, aligning with national priorities to shift toward preventative care. However, operationalising such interventions will require addressing resource and capacity challenges within the NHS. Further innovation to enhance early identification and preventive action should target women approaching menopause.

## Supplementary material

10.1136/bmjph-2025-003892 online supplemental file 1

## Data Availability

Data are available on reasonable request.
