# Supplementary material for: Perceptions of osteoporosis risk assessment during menopause: a qualitative focus group study of patients and healthcare professionals in the UK
Source: BMJ Public Health. 2026 Aug 11;4(3):e003892. doi: 10.1136/bmjph-2025-003892 (PMC13475154; doi:10.1136/bmjph-2025-003892)
Supplement: Supplementary file 1 [file bmjph-4-3-s001.docx]

**Patients**

**Workshop 1 - Problems**

**Tell me about your experience with osteoporosis diagnosis**

- How long ago were you diagnosed with osteoporosis? (if you have)
- How long did it take for you to be diagnosed?
- How does osteoporosis affect your daily life (if it does)
- Any major positives / negatives

**Any reflections on the process of osteoporosis diagnosis**

- waiting times
- diagnosis
- treatment
- support for osteoporosis

Anything else you would like us to know about your experience of osteoporosis?

**Workshop 2 - Solutions**

Please introduce yourself and say a bit about your osteoporosis journey

What do you think about devices that diagnose osteoporosis?

What do you think about the potential to be diagnosed in different settings:

- In a pharmacy
- By post
- In a health hub
- In a care home
- In a gym

Who would benefit from diagnosis, who would be discriminated against/

Do you think people would pay privately for osteoporosis testing?

Anything else you would like us to know about your experience of osteoporosis?

**Healthcare Professionals**

**Workshop 1 - Problems**

**Tell me about your experience with osteoporosis diagnosis**

- Where do you work / what type of patients do you work with?
- What is your experience of diagnosing / treating / supporting people with osteoporosis?
- How do you think a patient is currently identified for testing?
- What do you think about current methods of diagnosis?
- How does osteoporosis affect patients' daily lives in your experience?
- Any major positives / negative examples to share

**Any reflections on the process of osteoperosis diagnosis**

- waiting times
- diagnosis
- treatment
- support for osteoporosis

**Future Changes**

- Do you think diagnosis could happen outside GP/hospital?
- Which groups should be targeted for diagnosis?
- How might your work in this area change in the future?

Anything further reflections on your experience of osteoporosis?

**Workshop 2 - Solutions**

Please introduce yourself and say a bit about your professional osteoporosis experience

What do you think about devices that diagnose osteoporosis?

What do you think about the potential to be diagnosed in different settings:

- In a pharmacy
- By post
- In a health hub
- In a care home
- In a gym

Who would benefit from diagnosis, who would be discriminated against?

Do you think people would pay privately for osteoporosis testing?

Anything else you would like us to know about your experience of osteoporosis?
